# Analysis of deaths following yeast-derived hepatitis B vaccination of infants, China, January 2013 to December 2020

**DOI:** 10.3389/fpubh.2023.1170483

**Published:** 2023-06-16

**Authors:** Sihui Zhang, Tianshuo Zhao, Linyi Chen, Mingzhu Xie, Qing-Bin Lu, Juan Du, Jing Zeng, Ninghua Huang, Yaqiong Liu, Chao Wang, Fuqiang Cui

**Affiliations:** ^1^Department of Epidemiology and Biostatistics, School of Public Health, Peking University, Beijing, China; ^2^Department of Laboratorial Science and Technology & Vaccine Research Center, School of Public Health, Peking University, Beijing, China

**Keywords:** hepatitis B vaccine, deaths following immunization, infants, abnormal reactions, coincidental events

## Abstract

In China, adverse events following immunization (AEFI) are reported by the China AEFI Surveillance System (CNAEFIS). Serious AEFI, including deaths, are mandatorily reported and are evaluated for causality by province-or prefecture-level panels of experts. Yeast-derived HepB is the most widely used HepB in China for infants. However, the information about the death of infants caused by HepB is unclear. The CNAEFIS data on deaths following HepB from 2013 to 2020 were used for analyses. Descriptive analysis of epidemiologic characteristics was used to report death cases following HepB. We used administered doses to calculate denominators to estimate the risk of death after vaccination. During 2013–2020, there were 161 deaths following the administration of 173 million doses of HepB, for an overall incidence of 0.9 deaths per million doses. One hundred fifty-seven deaths were categorized as coincidental, and four deaths were accompanied by an abnormal reaction determined to be unrelated to the cause of death. The most common causes of death were neonatal pneumonia and foreign body asphyxia. These data provide reliable evidence on the safety of HepB among infants in China and can enhance public confidence in HepB immunization. To ensure public confidence in infants’ HepB vaccination, monitoring and scientifically evaluating AEFI-related deaths of HepB is necessary.

## 1. Introduction

China has had a longstanding health problem with chronic hepatitis B (CHB). Historically, approximately 10% of the population lived with CHB, placing them at high risk of cirrhosis and liver cancer as adults (1). Mother-to-child transmission (MTCT) is the main cause of hepatitis B virus (HBV) transmission among endemic populations (2, 3). To interrupt HBV MTCT, China began using plasma-derived HBV vaccine (HepB) in 1985 in pilot projects. In 1992, the Chinese firm Biokangtai (BKT) received yeast-derived recombinant HepB for licensure and production in China through a technology transfer agreement with Merck Vaccines. That year, China implemented universal HepB vaccination for infants with BKT HepB (4).

Passive-active immunoprophylaxis, strategic use of HepB vaccines and hepatitis B immunoglobulin (HBIG) in newborn infants of HBV-infected mothers was crucial as the first step in eliminating HBV infection, decreasing the HBsAg prevalence among children younger than 5 years by 97%, from 9.7% in the pre-vaccine era to 0.3% in 2014 (5, 6). This effort averted 120 million infections, 28 million CHB cases, and 5 million deaths by 2014, making the current generation of children free from hepatitis B and protected for life (5). Because many women of childbearing age were born before the ready access to HepB, hundreds of thousands of HBV-exposed infants are born each year in China, making continuing and strengthening the HBV MTCT program imperative for decades to come.

Successes of China’s HBV prevention program were threatened in late 2012 when national, international, and social media reported infant deaths following the HepB administration in Hunan, China, implying that HepB caused the deaths (7). HepB license was suspended during a month-long investigation that exonerated the vaccine and its production. The 17 deaths were evaluated by an independent panel of pediatricians that determined none of the deaths were caused by the HepB. The HepB license was restored, but a decrease in confidence caused the birth-dose coverage among HBV-exposed infants to decline six percentage points over a 4-week period before recovering to its high pre-event coverage (7).

In China, adverse events following immunization (AEFI) are reported by healthcare providers to the China AEFI Surveillance System (CNAEFIS), a passive surveillance system jointly operated by the National Medical Products Administration (NMPA) and the Chinese Center for Disease Control and Prevention (8–10). Serious AEFI, including deaths, are mandatorily reported and are evaluated for causality by province-or prefecture-level panels of experts.

HepB is administered in the neonatal period – a time of life associated with a significant background death rate, primarily from congenital anomalies, prematurity, and infection. To maintain confidence in HepB vaccination, periodically evaluating the epidemiology of deaths following HepB administration is crucial. Therefore, this study evaluated the deaths following the HepB vaccination during 2013 to 2020 in China, right after the “2012 HepB vaccine event.”

## 2. Materials and methods

### 2.1. Data collection

According to CNAEFIS, the AEFI (including reports of deaths) are classified into one of the following categories (9): (1) vaccine reactions or vaccine-related reactions, defined as unexpected adverse reactions or reactions unrelated to the intended vaccination purpose that occur after standard vaccination of vaccine products, including common and rare adverse reactions, (2) vaccine quality reactions, defined as damage to tissues and organs caused by substandard vaccine quality, which damages the function of the vaccinated person. Unqualified quality refers to issues with the vaccine’s product line, purity, production process, additives (excipients), external factors, and issues where inspection and control do not comply with national vaccine production plans or standards, (3) program errors or immunization errors, defined as damage to tissues or organs and damage to the function of vaccine recipients due to violations of standard operating procedures, vaccination procedures, and vaccine usage guidelines, (4) coincidental events are that the vaccinators were in the incubation or preclinical stage of a certain disease, and the onset of the disease coincides with the time of vaccination. Inherent characteristics of vaccines do not cause coincidental events, and (5) psychogenic reactions or immunization anxiety-related reactions mean that individual or group reactions occur during or after vaccination due to the psychological reactions of the recipient. Inherent characteristics of vaccines do not cause the psychogenic reactions.

Through the BKT request, all reports of AEFI in infants following the HepB administration reported to CNAEFIS from January 2013 to December 2020 were obtained. The records included age and sex, clinical symptoms, time intervals from vaccination to symptom onset, clinical diagnoses, and vaccines co-administered with HepB. Reports also included results of causality assessments conducted by provincial and prefecture-level expert panels.

### 2.2. Data analysis

Descriptive analysis of epidemiologic characteristics was used to report death cases following HepB. The individual characteristics were presented as numbers and percentages. Yearly administered HepB doses were collected from vaccination clinics during the study period as the denominator to estimate the risk of deaths following HepB. The death rates were calculated per million administered vaccination doses. Stata 16.0 (Stata Corp LP, College Station, TX) was used for all statistical analyses.

## 3. Results

From January 2013 to December 2020, there were 15,671 adverse events recorded among infants when 173,555,759 doses of HepB were released to the market. One hundred sixty-one deaths were recorded. Among the deaths, 156 (97.5%) were determined to have been coincidental deaths unassociated with any adverse reaction. A total of four (2.5%) deaths were accompanied with an abnormal reaction, unrelated to the death. None of the deaths were classified as vaccine quality defect-related, program error, or immunization anxiety-related events.

### 3.1. Characteristics of deaths accompanied by abnormal reactions

The four deaths accompanied by abnormal vaccine reactions are shown in Table 1. Of four deaths, two were girls. The age of vaccination ranged from 36 to 126 days. The interval from vaccination to clinical onset ranged from 1 to 4 days. A total of three deaths followed the second dose of HepB, and one followed the third dose of HepB. Another three deaths followed HepB co-administered with other vaccines – two with diphtheria, tetanus and a cellular pertussis combined vaccine (DPaT) and one with the polio vaccine. Another two deaths were associated with fever; none had redness or induration local reactions. The causes of the deaths included foreign body asphyxia, congenital heart disease, intracranial hemorrhage or edema, accompanied by anaphylactic reaction, anaphylactic shock, and epilepsy, for three deaths, respectively. Another one was indeterminate due to no clear clinical diagnostic record.

**Table 1:** Deaths accompanied with abnormal reactions following HepB immunization, China, 2013–2020 (*n* = 4).

Case no.	Date of birth	Sex	Birth to vaccination date	Doses	Other vaccines	Onset time of reaction, d	Fever/redness/induration	Abnormal reaction	Clinical diagnosis
1	2015/10/23	Female	126	2	DPaT	1	None	Anaphylaxis	Foreign body asphyxia
2	2016/11/9	Male	181	3	DPaT	1	Fever 38.5°C	Anaphylactic shock	Congenital heart disease
3	2016/6/16	Female	36	2	–	1	Fever 39.5°C	Not recorded	Indeterminate
4	2020/8/21	Male	65	2	polio	4	None	Epilepsy	Intracranial hemorrhage and edema

DPaT, diphtheria, pertussis, and tetanus mixed vaccine; Polio, polio vaccine.

### 3.2. Characteristics of deaths accompanied by no adverse reactions

**Figure 1:**
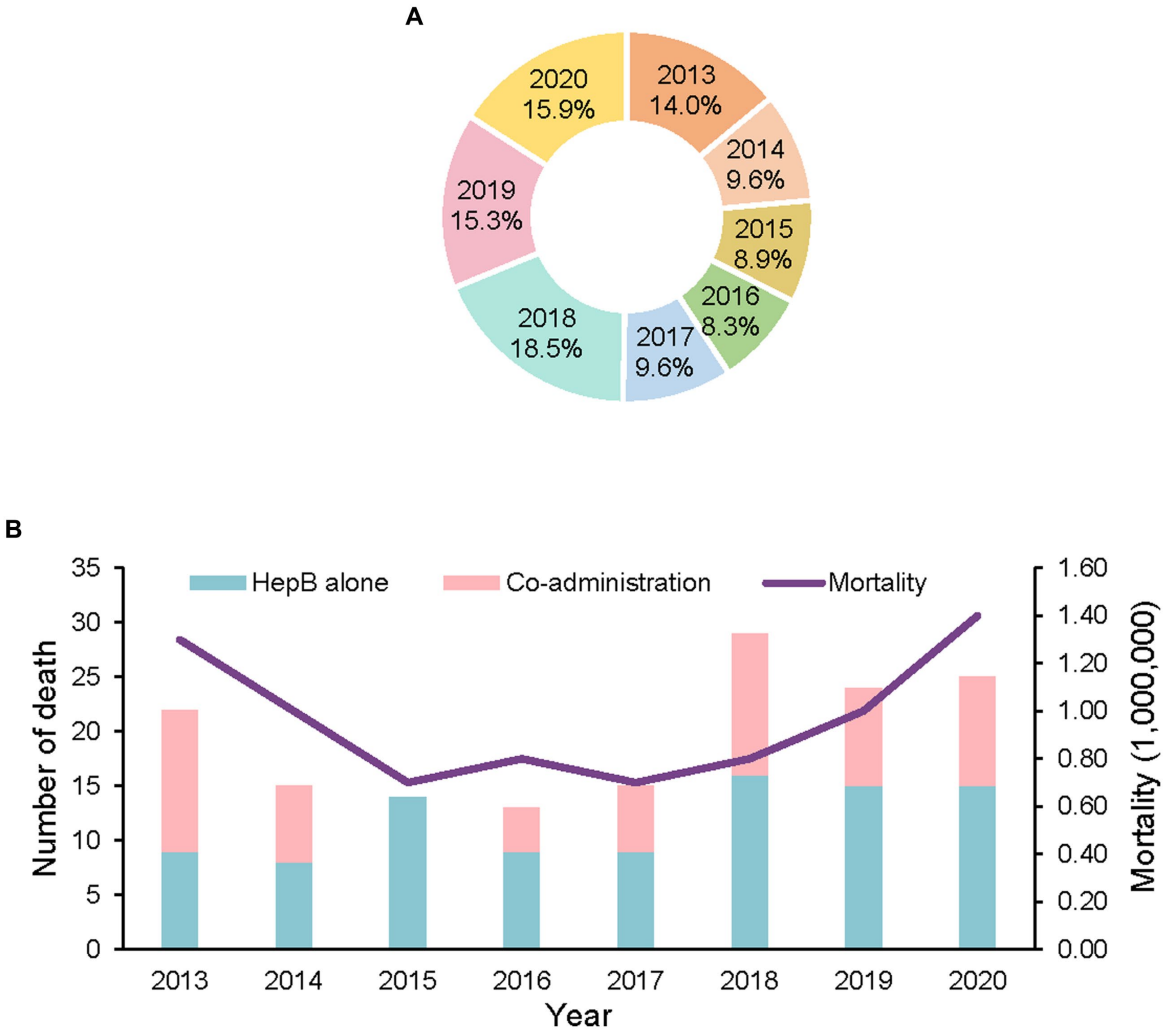
Reports of deaths with no adverse reaction following HepB by year, 2013–2020. **(A)** the proportion of deaths by year, 2013–2020. **(B)** number of deaths and mortality by year, 2013–2020.

There were 157 deaths reported in which there were no adverse vaccine reactions. The overall incidence of death was 0.9 per million doses, with annual incidences ranging from 0.7/million to 1.4/ million doses. The highest annual incidence occurred in 2020. During the eight-year study period, 18.5% of the deaths were reported in 2018. Surveillance sensitivity increased by 2018 when death-case reports began to increase (Figure 1). Approximately half (49.7%) of the deaths were reported during 2018–2020.

Table 2 shows the characteristics of these deaths. Among the deaths, 98 (62.4%) were boys; 47 (29.94%) were below one-month-old; 74 (47.13%) were 1–2 months old; 36 (22.93%) were above 2 months. 43 (27.4%) followed the first dose of HepB, 100 (63.7%) followed the second dose of HepB, and 14 (8.9%) followed the third dose of HepB. Sixty-two (39.5%) deaths followed the co-administration of one or two other vaccines, among which the birth dose of the BCG vaccine was the most frequently co-administered vaccine (accompanied by 40 deaths). Four deaths followed the co-administration of two vaccines. The most common causes of death were neonatal pneumonia (36, 28.6%), foreign body asphyxia (17, 10.6%), congenital heart disease (12, 7.5%), sudden infant death syndrome (10, 6.2%), and neonatal respiratory distress syndrome (6, 3.7%). Forty-six deaths were indeterminate, and most (44, 95.7%) died before being admitted to a hospital.

**Table 2:** Death reports with no adverse reaction following HepB immunization, China, 2013–2020 (*n* = 157).

Demographic		Number	Proportion (%)
Sex	Male	98	62.4
	Female	59	37.6
Month age at vaccine, months	<1	47	29.9
	1	74	47.1
	≥2	36	22.9
Dose	1	43	27.4
	2	100	63.7
	3	14	8.9
Co-administration	None	95	60.5
	One more vaccine	63	39.5
	BCG	40	25.5
	Polio	10	6.4
	DPT	5	3.2
	MenA/C	5	3.2
	Influenza vaccine	2	1.3
	Two more vaccines	4	2.5
	DPaT+ Polio	3	1.9
	MenA/C + Polio	1	0.6
Clinical diagnosis	Indeterminate cause of death	46	28.6
	Neonatal pneumonia	36	22.4
	Foreign body asphyxia	17	10.6
	Congenital heart diseases	12	7.5
	Sudden infant death syndrome	10	6.2
	Neonatal respiratory distress syndrome	6	3.7
	Intracranial hemorrhage and edema	5	3.1
	Pulmonary hemorrhage	3	1.9
	Severe diarrhea with dehydration	3	1.9
	Coagulation disorder	2	1.2
	Hemorrhagic necrotizing enteritis	2	1.2
	Neonatal cyanosis	2	1.2
	Hyperammonemia	2	1.2
	Other	11	7.0
Total		157	

BCG, Bacille Calmette-Guerin; Polio, polio vaccine; DPaT, diphtheria, pertussis, and tetanus mixed vaccine; MenA/C, meningococcal A/C vaccine.

## 4. Discussion

Our study revealed that between 2013 and 2020, over 173 million doses of BKT HepB were released to the market in China. During this time, 161 infant deaths were reported to CNAEFIS following the administration of HepB. Abnormal adverse reactions accompanied four deaths, and none of them were the cause of death; 157 were determined to be coincidental deaths unassociated with adverse reactions. The overall incidence of 0.9 deaths per million doses, with no vaccine-caused deaths, is in line with results reported by the Vaccine Adverse Event Reporting System in the United States (11) and is similar to another study in China (12). An earlier study by Niu and colleagues reported that the incidence of death following HepB ranged from 1.0/million to 8.6/million doses depending on age group and the brand of HepB (13).

Of the four deaths with abnormal reactions, two were girls following the second dose of HepB after birth. Two cases were reported following the co-administration of DPaT and one following the polio vaccine. It is suggested that co-administration may increase the possibility of AEFI but may not be directly related to the cause of death (12). The death from foreign body asphyxia had an accompanying anaphylactic reaction caused by either the vaccine itself or by excipients contained in the vaccine (14–17). The anaphylactic reaction was not determined to be the cause of death. One case died of intracranial hemorrhage and edema, and the abnormal reaction was classified as epilepsy associated with brain lesions or caused by a complex genetic predisposition (18–20). Still, there was no evidence of causality with vaccination. Another case was diagnosed with congenital heart disease, whose abnormal reaction was anaphylactic shock, an allergic reaction with cardiovascular collapse, which might be caused by food, drugs, or hymenoptera stings (21, 22). Anaphylactic shock may be a contributing factor to death (22). The current evidence was too limited to draw affirmative conclusions regarding associations between death and the HepB vaccination.

Public awareness of CNAEFIS has been suggested to lead to increasing reporting of adverse events after 2013 (7). The Chinese Vaccine Circulation Law was enacted in 2019 and may have increased surveillance sensitivity of AEFI-associated deaths through increased awareness of CNAEFIS (23). As for deaths accompanied by no adverse reactions, this review found that death reports increased sufficiently 1 year before the formal implementation of the law when draft legislation was being vetted publicly. The higher proportion of male deaths is consistent with a previous study (12). More deaths occurred following the second dose of HepB, and the BCG vaccine was reported as the most frequently co-administered vaccine, consistent with the previous research (12). Analysis of other deaths reported to CNAEFIS showed that although co-administration may increase the possibility of AEFI, it was unrelated to the cause of death (12).

The 2012 HepB event raised widespread public concern (24, 25). The 17 deaths were evaluated by an independent panel of pediatricians who found similar causes of death to our findings (24, 25). Considering coincidental infant deaths will always happen since the background neonatal mortality rate is non-zero, comprehensive and systematic approaches for preparing and responding to adverse events following HepB should be developed and used when needed (26).

Infants are vaccinated with HepB within 24 h after birth given two more doses in infancy. This post-exposure prevention strategy can prevent 95% of the vertical transmission of HBV and provide lifelong protection against HBV (27). We believe that our study provides valuable information in enhancing vaccine confidence.

A limitation of our study was that autopsies are not routinely conducted in the death analyses, adding imprecision to the causes of death.

## 5. Conclusion

China’s program to prevent mother-to-child transmission of HBV is a fundamental child health program. Our review provides assurance of the safety of BKT HepB and reinforces the necessity of conducting scientific evaluations of serious AEFI reports to maintain public confidence in vaccines and immunization.

## Data availability statement

The raw data supporting the conclusions of this article will be made available by the authors, without undue reservation.

## Ethics statement

This study was approved by Peking University Institutional Review Board (IRB00001052-21008). Written informed consent from the participants’ legal guardian/next of kin was not required to participate in this study.

## Author contributions

FC and CW: conceptualization. SZ, TZ, LC, and MX: formal analysis. FC: funding acquisition. SZ: writing – original draft preparation. FC, Q-BL, JD, JZ, NH, YL, and CW: writing – review and editing. All authors contributed to the article and approved the submitted version.

## Funding

This work was supported by the National Key Research and Development Program of China (Grant number 2021YFC2301604), Fundamental Research Funds for the Central Universities and Peking University Health Science Center (Grant number BMU2022XY030).

## Conflict of interest

The authors declare that the research was conducted in the absence of any commercial or financial relationships that could be construed as a potential conflict of interest.

## Publisher’s note

All claims expressed in this article are solely those of the authors and do not necessarily represent those of their affiliated organizations, or those of the publisher, the editors and the reviewers. Any product that may be evaluated in this article, or claim that may be made by its manufacturer, is not guaranteed or endorsed by the publisher.

## References

[1] Zheng Y Wu J Ding C Xu KJ Yang SG Li LJ. Disease burden of chronic hepatitis B and complications in China from 2006 to 2050: an individual-based modeling study. Virol J. (2020) 17:132. doi: 10.1186/s12985-020-01393-z, PMID: 32859216 PMC7455911

[2] Cheung KW Lao TT. Hepatitis B – vertical transmission and the prevention of mother-to-child transmission. Best Pract Res Clin Obstet Gynaecol. (2020) 68:78–88. doi: 10.1016/j.bpobgyn.2020.02.014 32249130

[3] Fan R Yin X Liu Z Liu Z Lau G Hou J. A hepatitis B-free generation in China: from dream to reality. Lancet Infect Dis. (2016) 16:1103–5. doi: 10.1016/S1473-3099(16)30327-9, PMID: 27676339

[4] Yuan XJ Wu F Luo D. (2022). Kangtai biotechnology: 30 years of vaccination, original intention unchanged. Shenzhen Special Economic Zone News; 2022-12-01(A08).

[5] Cui F Shen L Li L Wang H Wang F Bi S. Prevention of chronic hepatitis B after 3 decades of escalating vaccination policy, China. Emerg Infect Dis. (2017) 23:765–72. doi: 10.3201/eid2305.161477, PMID: 28418296 PMC5403029

[6] Tang J Luo YQ Zhou YH. Elimination of hepatitis B virus infection in children: experience and challenge in China. Chin Med J. (2021) 134:2818–24. doi: 10.1097/CM9.0000000000001791, PMID: 34636773 PMC8667976

[7] Meina L Xiaodong L Lulu Z. Hepatitis B vaccine adverse events in China: risk control and regulation. Hum Vaccin Immunother. (2014) 10:2992–3. doi: 10.4161/21645515.2014.971643, PMID: 25483642 PMC5443087

[8] Guo B Page A Wang H Taylor R McIntyre P. Systematic review of reporting rates of adverse events following immunization: an international comparison of post-marketing surveillance programs with reference to China. Vaccine. (2013) 31:603–17. doi: 10.1016/j.vaccine.2012.11.051, PMID: 23200940

[9] Liu D Wu W Li K Xu D Ye J Li L. Surveillance of adverse events following immunization in China: past, present, and future. Vaccine. (2015) 33:4041–6. doi: 10.1016/j.vaccine.2015.04.060, PMID: 25936727

[10] Wu WD Liu DW Wu BB. Analysis on the surveillance of adverse events following immunization in China, 2007–2008. Zhongguo Yi Miao He Mian Yi. (2009) 15:481, 538–90. PMID: 20518320

[11] Niu MT Salive ME Ellenberg SS. Neonatal deaths after hepatitis B vaccine – the vaccine adverse event reporting system, 1991–1998. Arch Pediatr Adolesc Med. (1999) 153:1279–82. doi: 10.1001/archpedi.153.12.1279, PMID: 10591306

[12] Wu W Liu D Nuorti JP Li K Xu D Ye J. Deaths reported to national surveillance for adverse events following immunization in China, 2010–2015. Vaccine. (2019) 37:1182–7. doi: 10.1016/j.vaccine.2019.01.009, PMID: 30709723

[13] Niu MT Rhodes P Salive M Lively T Davis DM Black S. Comparative safety of two recombinant hepatitis B vaccines in children: data from the vaccine adverse event reporting system (VAERS) and vaccine safety datalink (VSD). J Clin Epidemiol. (1998) 51:503–10. doi: 10.1016/S0895-4356(98)00014-6, PMID: 9635999

[14] Kounis NG Koniari I de Gregorio C Velissaris D Petalas K Brinia A. Allergic reactions to current available COVID-19 vaccinations: pathophysiology, causality, and therapeutic considerations. Vaccines (Basel). (2021) 9:221. doi: 10.3390/vaccines9030221 33807579 PMC7999280

[15] Kelso JM Greenhawt MJ Li JT Nicklas RA Bernstein DI Blessing-Moore J. Adverse reactions to vaccines practice parameter 2012 update. J Allergy Clin Immunol. (2012) 130:25–43. doi: 10.1016/j.jaci.2012.04.003, PMID: 22608573

[16] Su JR Moro PL Ng CS Lewis PW Said MA Cano MV. Anaphylaxis after vaccination reported to the vaccine adverse event reporting system, 1990–2016. J Allergy Clin Immunol. (2019) 143:1465–73. doi: 10.1016/j.jaci.2018.12.1003, PMID: 30654049 PMC6580415

[17] McNeil MM Weintraub ES Duffy J Sukumaran L Jacobsen SJ Klein NP. Risk of anaphylaxis after vaccination in children and adults. J Allergy Clin Immunol. (2016) 137:868–78. doi: 10.1016/j.jaci.2015.07.048, PMID: 26452420 PMC4783279

[18] Berg AT Coryell J Saneto RP Grinspan ZM Alexander JJ Kekis M. Early-life epilepsies and the emerging role of genetic testing. JAMA Pediatr. (2017) 171:863–71. doi: 10.1001/jamapediatrics.2017.1743, PMID: 28759667 PMC5710404

[19] Gutierrez-Delicado E Serratosa JM. Genetics of the epilepsies. Curr Opin Neurol. (2004) 17:147–53. doi: 10.1097/00019052-200404000-00011 15021241

[20] Weckhuysen S Marsan E Lambrecq V Marchal C Morin-Brureau M An-Gourfinkel I. Involvement of GATOR complex genes in familial focal epilepsies and focal cortical dysplasia. Epilepsia. (2016) 57:994–1003. doi: 10.1111/epi.13391, PMID: 27173016

[21] Wawrzyniak P Akdis CA Finkelman FD Rothenberg ME. Advances and highlights in mechanisms of allergic disease in 2015. J Allergy Clin Immunol. (2016) 137:1681–96. doi: 10.1016/j.jaci.2016.02.010, PMID: 27090934

[22] Simons FE. 9. Anaphylaxis. J Allergy Clin Immunol. (2008) 121:S402–7; quiz S20. doi: 10.1016/j.jaci.2007.08.061 18241691

[23] Wang C Huang NH Lu QB Black S Liang XF Cui FQ. Change in adverse event reporting following immunization of hepatitis B vaccine among infants between 2013 to 2020 before and after the vaccine administration law in China. Front Immunol. 13:956473. doi: 10.3389/fimmu.2022.956473 PMC9561938 36248783

[24] Hendriks J Liang Y Zeng B. China's emerging vaccine industry. Hum Vaccin. (2010) 6:602–7. doi: 10.4161/hv.6.7.11933 20523120

[25] Yu WZ Liu DW Zheng JS Liu YM An ZJ Rodewald L. Loss of confidence in vaccines following media reports of infant deaths after hepatitis B vaccination in China. Int J Epidemiol. (2016) 45:441–9. doi: 10.1093/ije/dyv349, PMID: 27174834

[26] Gidudu JF Shaum A Habersaat K Wilhelm E Woodring J Mast E. An approach for preparing and responding to adverse events following immunization reported after hepatitis B vaccine birth dose administration. Vaccine. (2020) 38:7728–40. doi: 10.1016/j.vaccine.2019.07.041, PMID: 31337590 PMC10335103

[27] Liang XF Bi SL Yang WZ Wang LD Cui G Cui FQ. Epidemiological serosurvey of hepatitis B in China-declining HBV prevalence due to hepatitis B vaccination. Vaccine. (2009) 27:6550–7. doi: 10.1016/j.vaccine.2009.08.048, PMID: 19729084

